# Early weight-bearing after anterior cruciate ligament reconstruction with hamstring grafts induce femoral bone tunnel enlargement: a prospective clinical and radiographic study

**DOI:** 10.1186/s12891-019-2653-6

**Published:** 2019-06-04

**Authors:** Takuya Tajima, Nami Yamaguchi, Makoto Nagasawa, Yudai Morita, Yoshihiro Nakamura, Etsuo Chosa

**Affiliations:** Division of Orthopedic Surgery, Department of Medicine of Sensory and Motor Organs, Faculty of Medicine, University of Miyazaki, 5200 Kihara, Kiyotake, Miyazaki, 889-1692 Japan

**Keywords:** Anterior cruciate ligament, Tunnel enlargement, Hamstring, Non-weight-bearing

## Abstract

**Background:**

Bone tunnel enlargement following primary anterior cruciate ligament (ACL) reconstruction with soft tissue graft might be a severe disadvantage for revision surgery. The postoperative rehabilitation protocol including the non-weight-bearing periods were different depending on the surgeon or institute. To determine the relationship between femoral bone tunnel enlargement and the postoperative non-weight-bearing period after double-bundle ACL reconstruction with hamstring grafts.

**Methods:**

Forty-two patients who underwent primary double-bundle ACL reconstruction with hamstring grafts were divided into two postoperative non-weight-bearing protocol groups: 1-week non-weight-bearing postoperatively (group A, *n* = 19); and 2-week non-weight-bearing (group B, *n* = 18). Five cases were excluded due to additional knee injury, pregnancy, and lost to follow-up.

Bone tunnel enlargement was evaluated by computed digital radiographs (anteroposterior (A-P) and lateral views) taken on the first postoperative day and at 12 months. Each tunnel diameter was shown as a percentage to the maximum joint width of the proximal tibia in the A-P view, or a percentage of the maximum diameter of the patella in the lateral view. To determine the incidence of tunnel enlargement, percentage diameter changes of more than 10% were defined as an enlarged tunnel. The magnitude of tunnel enlargement and the standard clinical evaluation were also evaluated.

**Results:**

There were no significant differences between groups in the incidences of anteromedial and posterolateral bone tunnel enlargement, both in the A-P and lateral views (2 × 2 Chi-squared test). The magnitude of femoral posterolateral bone tunnel enlargement was significantly greater in group A in the A-P view (*p* = 0.01) and lateral view (*p* = 0.03) (Mann Whitney U-test). Twelve months after surgery, the Lysholm score and Tegner activity level scale were not significantly different between the groups.

**Conclusions:**

This prospective, clinical and radiographical study showed that early weight-bearing protocol after double-bundle ACL reconstruction with hamstring grafts might have the potential risk of significant postoperative femoral bone tunnel enlargement of the posterolateral bundle. There was no significant difference in clinical outcomes by postoperative non-weight-bearing period. To reduce and prevent the femoral bone tunnel enlargement, the comprehensive management could be considered and required to establish the suitable early stage rehabilitation protocol after surgery.

**Trial registration:**

Trial registration number; UMIN000036212.

Scientific title: Prospective comparisons of femoral tunnel enlargement with two different postoperative non weight bearing periods after double-bundle anterior cruciate ligament reconstruction with hamstring grafts.

Registered date: 15 Mar 2019 (retrospectively registered).

## Background

Recently, ACL reconstruction procedures have developed with progress in basic research, surgical devices, and surgical techniques [1]. ACL reconstruction has become one of the commonly performed procedures for ACL-deficient athletes. Previously, the incident rate of ACL rupture has been reported to be between 37 and 61% per person-years, and there were estimates that over 200,000 new ACL injuries occur annually in United States [2–4]. Many surgical procedures for ACL reconstruction and good clinical outcomes have been reported [5–7].

Rotational laxity due to significant translation of the lateral compartment in single-bundle compared with double-bundle ACL reconstruction has been reported [8–10]. On the other hand, the anatomically ACL-reconstructed knee showed satisfactory restoration of rotator laxity, as measured by an electromagnetic measurement system, independent of the surgical procedure [11]. However, unfortunately, failure and recurrent instability rate of ACL reconstruction have been reported between 10 and 15%, leading to a large number of revision ACL reconstruction [12, 13].

For graft selection, bone-patellar tendon-bone (BTB) grafts and soft tissue grafts including hamstring grafts are frequently used. In cases of ACL reconstruction with soft tissue grafts, postoperative bone tunnel enlargement has been reported [14–21]. Bone tunnel enlargement following primary ACL reconstruction might be a severe disadvantage for revision surgery; even well-positioned graft in widened tunnels could present a significant challenge during revision ACL reconstruction. Tunnel enlargement and resultant bone loss and poor fixation including tunnel wall-graft incorporation significantly increase the difficulty of revision ACL reconstruction [22]. An additionally bone grafting, primary or staged, was often required for the previous enlarged bone tunnel have been reported [22, 23].

Postoperative rehabilitation protocols including the immobilization period and the non-weight-bearing period in the acute phase after surgery depend on each surgeon or institute, and they are not clearly standardized. Post-operative rehabilitation has been implicated in bone tunnel enlargement, with some studies suggesting a decrease in graft micromotion and tunnel enlargement with nonaggressive rehabilitation [24]. Early aggressive rehabilitation protocols may contribute to bone tunnel enlargement as it subjects the graft-bone interface to early stress before biological incorporation and ligamentization in complete [18, 24–26].

Previously, from clinical and radiographic findings of a prospective study, 1 week was recommended as a suitable postoperative immobilization period after ACL reconstruction with a hamstring autograft. A longer immobilization period, such as 2weeks, did not significantly reduce femoral bone tunnel enlargement, both in incidence and magnitude [27].

Lind et al. reported that bone tunnel enlargement measured by X-ray at 12 months after single-bundle ACL reconstruction with hamstring grafting and Endobutton CL was seen 46.2% in the anteroposterior (A-P) view and 38.5% in the lateral view [28]; Siebold reported 34% for AMB and 46% for PLB tunnel enlargement at 7 months postoperatively with magnetic resonance imaging (MRI) study [16]. Using the digital radiography for measuring the tunnel enlargement study, Kawaguchi et al. also reported that 22–36% of cases showed 24 months after double-bundle ACL reconstruction with hamstring grafting and Endobutton CL [29]. Taketomi et al. reported that bone tunnel enlargement after double bundle ACL reconstruction measure by computed tomography was shown 34.0% in horizontal, 28.2% in vertical of AMB, and 58.2% in horizontal, 73.4% in vertical view at 12 months after surgery [30].

However, the postoperative non-weight-bearing periods were not employed same protocol; some cases were allowed immediate full weight-bearing after surgery [28], and other cases required 2 days [30] or ~ 1 week [16] of non-weight-bearing postoperatively. Actually, the most suitable non-weight-bearing period protocol after double-bundle ACL reconstruction with hamstring graft was still obscure.

The purpose of this study was to determine the relationship between femoral bone tunnel enlargement, clinical outcomes and the postoperative non-weight-bearing periods after double-bundle ACL reconstruction with hamstring grafts. We conducted the present prospective study comparing two different postoperative non-weight-bearing periods by assessing the clinical and radiological results, including bone tunnel enlargement at 12 months following surgery. We hypothesized that a longer postoperative non-weight-bearing period protocol may contribute to prevent or reduce bone tunnel enlargement after surgery due to several biological or biomechanical events.

## Methods

### Patients

**Fig. 1:**
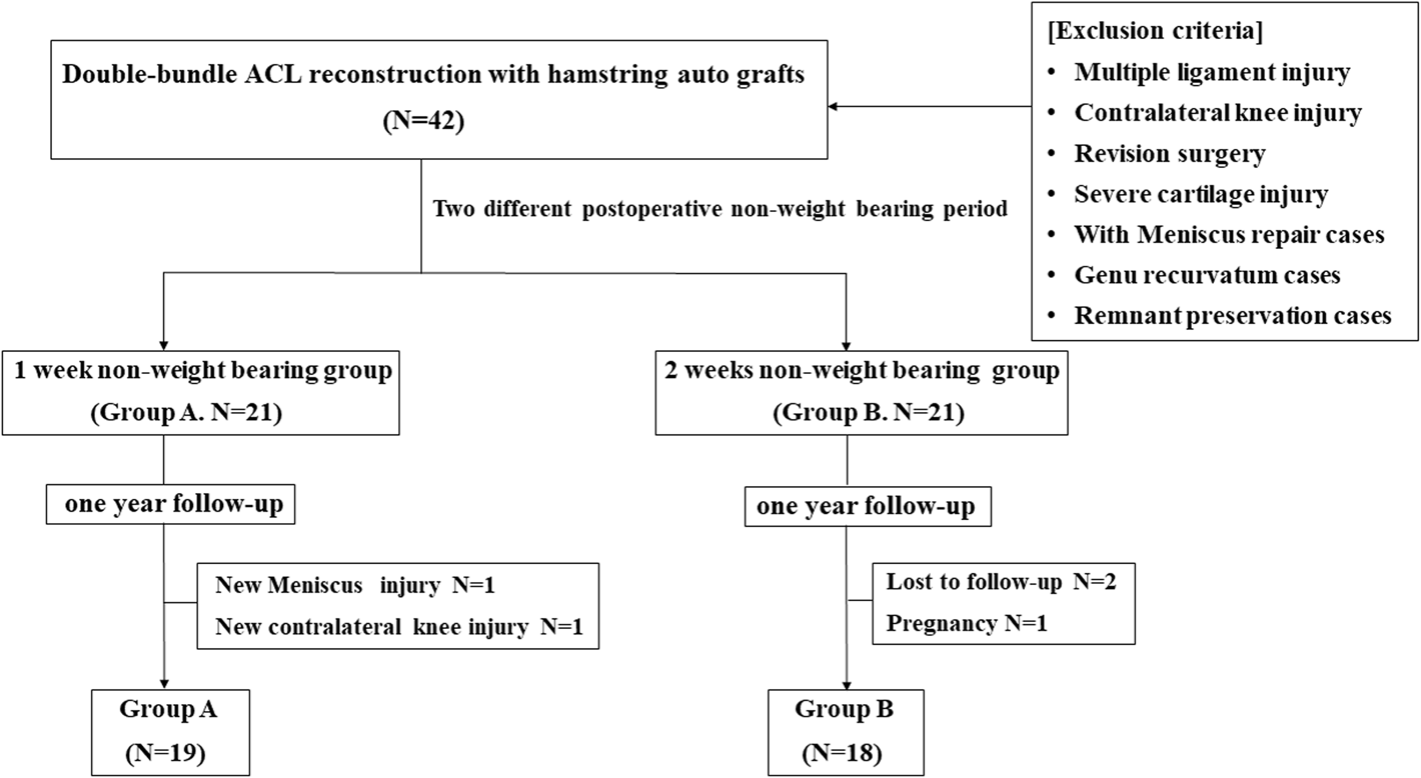
Participant flowchart

The present prospective, comparative clinical research was conducted in 2014, involving patients who underwent double-bundle ACL reconstruction with hamstring tendon autografts, under the same surgical technique and the same surgical devices at our institute. The authors were planning to examine the full clinical and radiological data available, such as general clinical scores, knee extension/flexion muscle strength measurements, and femoral bone tunnel enlargement measured by digital radiography of the knee 12 months postoperatively. Exclusion criteria of this study was established as multiple ligament injuries which was indicated the presence of abnormal posterior laxity or abnormal varus and valgus laxity, open growth plate cases, under 15 years old cases, the contralateral knee ligament injury cases, concomitant treatment for articular cartilage defects; not only osteochondral autologous transplantation, but also bone marrow stimulation procedures including micro fracture, remnant preserved augmentation cases and ACL reconstruction with meniscal repair cases, because these cases required a more longer non-weight-bearing protocol postoperatively in our institute. The genu recurvatum cases provided different gait pattern on knee angle or extension moments [31]. Therefore, genu recurvatum cases were excluded from the present study. Patients who did not want to take part were also excluded from the present study. Initially, total of 42 consecutive patients were enrolled in the present study; which were composed 21 historical control cases and 21 prospective cases (Fig. 1). During the follow-up period, two patients were lost to follow-up, one patient had another meniscal injury after primary ACL reconstruction, one patient had a contralateral ACL injury after primary surgery, and one patient could not complete the postoperative rehabilitation protocol due to pregnancy. Therefore, 37 patients met the inclusion criteria and were finally matched in this study (Fig. 1). These cases who were undergoing primary double-bundle ACL reconstruction with hamstring grafts were allocated into two different postoperative non-weight-bearing protocol groups; the allocation was conducted in a serial consecutive, not randomized, fashion: the first consecutive group of patients received our usual 1 week of non-weight-bearing protocol postoperatively (group A as historical control, *n* = 19; 9 males and 10 females); the second consecutive group of patients received 2 weeks of non-weight-bearing protocol (group B, *n* = 18; 7 males and 11 females). The patients’ characteristics are shown in Table 1.

**Table 1:** Characteristic of Patients

	Group A	Group B	*P value*
No. of cases	19	18	
Age (Range.years)	23.9 (15–40)	23.3 (15–48)	0.45
Gender (Male/Female)	19 (9/10)	18 (7/11)	0.6
Height (cm)	163.9 ± 7.7	162.7 ± 7.7	0.57
Weight (kg)	59.3 ± 8.7	61.3 ± 11.3	0.66
Cause of injury			
Sports activity	17	16	
Work or Accidents	2	2	
Graft size (diameter, mm)			
AMB	6.31 ± 0.3	.39 ± 0.4	0.81
PLB	5.19 ± 0.3	5.33 ± 0.4	0.41
Femoral tunnel length (mm)			
AMB	31.6 ± 5.2	33.9 ± 4.7	0.15
PLB	33.2 ± 3.4	34.7 ± 3.4	0.25
Graft length inside the femoral tunnel (mm)			
AMB	14.0 ± 1.9	15.2 ± 1.1	0.08
PLB	14.7 ± 2.2	14.6 ± 2.3	0.58

Values are expressed as mean ± standard deviation except cause of injury

Mann-Whitney U-test

*AMB* anteromedial bundle, *PLB* posterolateral bundle

### Surgical procedures

For every patient who was enrolled in this study, an arthroscopically diagnostic was performed to ensure that complete ACL tear was present and to examine for other possible findings; medial and lateral meniscal injuries or articular cartilage injuries in the knee. The anatomical double-bundle ACL reconstruction with hamstring procedure was described previously [27]. Femoral tunnels were created under trans-portal technique and inside-out fashion in all cases. The characteristics of graft and bone tunnel situation in detail are shown in Table 1. The EndoButton CL (Smith & Nephew, Andover, MA) and a double-spike plate system (Meira, Aichi, Japan) were used for graft fixation.

Under applying the tension of 20 N to both AM and PL grafts by assistant surgeon using the tensiometer, the grafts were fixed to the tibia with the knee positioned in 20° of flexion [32].

### Rehabilitation protocol

**Fig. 2:**
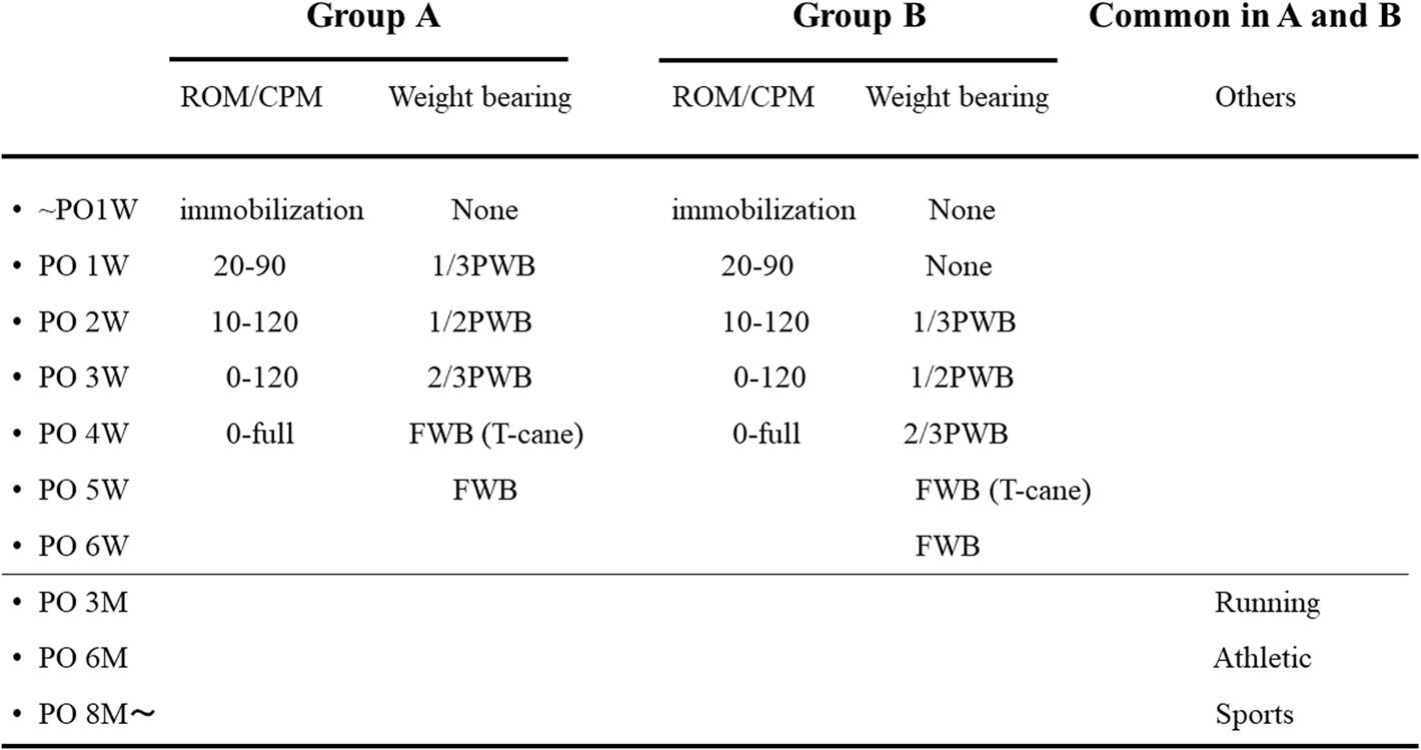
Rehabilitation protocol. Group A: partial weight-bearing was started at 1 week following surgery, with full weight-bearing at 4 weeks. Group B: partial weight-bearing was started at 2 weeks after surgery, with full weight-bearing at 5 weeks

In the operation room, the knee was immobilized with a brace with the knee positioned in 20° of flexion for both groups to maintain the same angle as during graft fixation at surgery to avoid excess stress on the grafts [32]. In this study, the knee functional brace was employed for prophylaxis of re-injury or in protecting the ACL graft following reconstruction [33]. After the protocol immobilization period, active and passive range of motion exercises were performed gradually with a functional brace (BREG X2K brace, BREG, CA, USA) (Fig. 2). In group A, partial weight-bearing was started at 1 week following surgery, with full weight-bearing at 4 weeks. For group B, partial weight-bearing was started at 2 weeks after surgery, with full weight-bearing at 5 weeks. All patients used the knee brace for the first 3 months after surgery. Jogging and running were allowed at 3 months postoperatively. Return to the athletic movement such as jumping or cutting actions were allowed at 6 months. and return to full sports activity was no sooner than 8 months after surgery (Fig. 2).

### Clinical evaluations

At 12 months postoperatively, follow-up clinical examinations were performed. The factors evaluated were the Lysholm score, the Tegnar activity level scale, and peak isokinetic quadriceps and hamstring torque at 60°/s measured with a Biodex-4 (Biodex Medical Systems Inc., Shirley, NY). Isokinetic peak torque values were presented as leg symmetry index, involve side/ non-involve side as 100(%). Side-to-side difference was also measured under an anterior tibial load of 134 N with a KNEELAX-3 arthrometer (Monitored Rehab Systems, Haarlem, The Netherlands). The knee range of motion measurement were evaluated by goniometer, especially extension lag was strictly measured both in supine and proneness position. Two experienced senior orthopedic surgeons performed these clinical examinations and collected the data.

### Radiographical evaluations

**Fig. 3:**
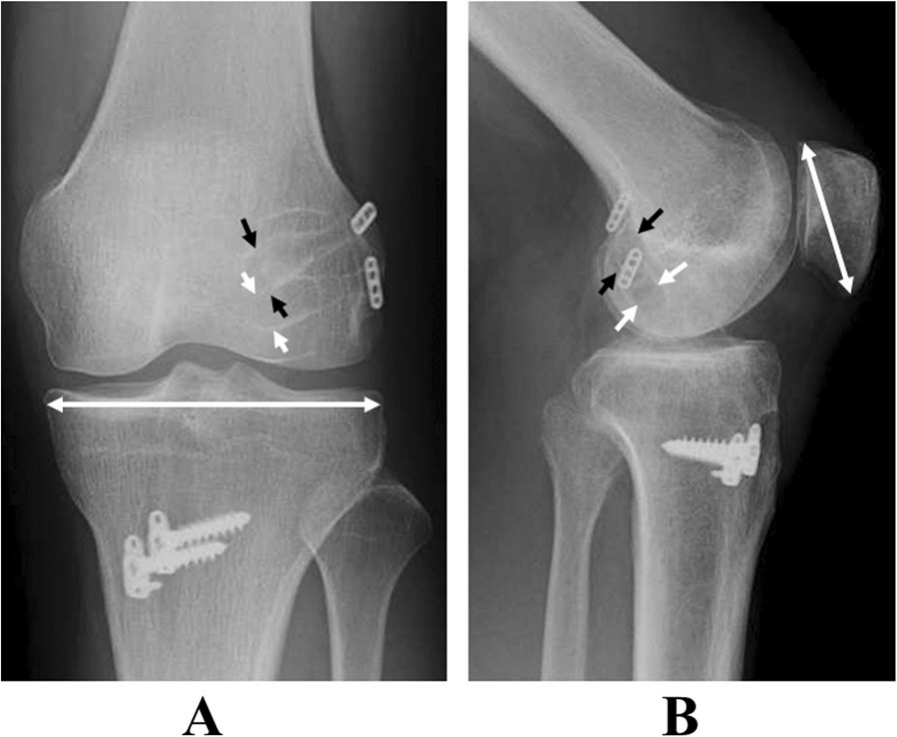
Computed digital radiographs of the knee with double-bundle ACL reconstruction. The two black arrows show the femoral outlet of the anteromedial tunnel, while the two white arrows show the femoral outlet of the posterolateral tunnel. **a** anteroposterior view 12 months after surgery. **b** lateral view 12 months after surgery

The radiological examinations were performed twice, on the first postoperative day and 12 months after surgery, to evaluate bone tunnel enlargement of both the AM and PL bundles in each group. The A-P and lateral views were taken by Computed digital radiographs (Fujifilm Corporation, Tokyo, Japan) to measure tunnel enlargement according to Webster et al. and Kawaguchi et al. (Fig. 3) [17, 29]. To determine the diameter of the bone tunnel, the tunnel wall margin was enhanced under computed operation with controlling contrast, intensity, and brightness of the image. To compare the femoral tunnel diameter in radiographs taken at the two different periods. Each diameter of bone tunnel was shown as a percentage to the maximum joint width of the proximal tibia in the anterior-posterior view, or a percentage to the maximum diameter of the patella in the lateral view. A percentage change between the two different periods was defined as percentage tunnel enlargement in diameter. To determine the incidence of tunnel enlargement, a percentage diameter change of more and less than 10% as an enlarged tunnel and a reduced tunnel, respectively [17, 27, 29]. The magnitude of percent femoral bone tunnel enlargement compared to the original size was also evaluated, with the original size considered as 100%. The incidence and magnitude of femoral bone tunnel enlargement were analyzed for the AMB and PLB separately. The tunnel enlargement grade was determined by three blinded, experienced orthopaedic surgeons.

### Statistical analysis

From the preliminary investigation, the standard deviation was 12, and difference was 12 (%). Based on a power of 80% and α of 0.05, the sample size required per group was calculated to be 16.7. Statistical comparisons between the two groups of the clinical results and the magnitude and incidence of bone tunnel enlargement were performed using the Mann Whitney U-test and the 2 × 2 Chi-squared test using the statistical software package BellCurve for Excel 2015 (Social Survey Research Information Co., Ltd., Tokyo, Japan). The level of significance was set at *P* < 0.05.

## Results

### Clinical results

Twelve months after surgery, clinical results including the Lysholm score (96.4 points in Group A, 97.1 points in Group B), Tegnar activity level scale (7.2 in both Groups), anterior laxity side-to-side difference (0.92 mm in Group A, and 0.45 mm in Group B), and muscle strength values in knee extension (93.1% in Group A, 93.0% in Group B) and flexion (89.3% in Group A, 94.3% in Group B) were satisfactory in both groups without significantly difference (Table 2). Knee extension deficiency of less than 5 degree was seen in one case (4 degree) in group A (5.2%) and three cases (1, 2 and 3 degree, respectively) in group B (16.7%), with no significant difference between the groups.

**Table 2:** Clinical results at 12 monthes postoperatively

	Group A (*n* = 19)	Group B (*n* = 18)	*P* Value
ACL reinjury (No. of cases) Lysholm score	0 96.4 ± 3.4	0 97.1 ± 3.1	0.65
Tegnar Activity Level Scale	7.2 ± 1.2	7.2 ± 1.3	0.89
Side-to-side difference			
Mean (mm)	0.92 ± 1.5	0.45 ± 1.1	0.12
< 2 mm (%)	85.7	94.7	
Quadriceps torque at 60°/sec			
presented as leg symmetry index, involve side/ non-involve side as 100(%)	93.1 ± 13.2	93.0 ± 16.7	0.6
Hamstring torque at 60°/sec			
presented as leg symmetry index, involve side/ non-involve side as 100(%)	89.3 ± 19.6	94.3 ± 15.3	0.15
knee extension deficiency			
< 5degree (cases.%)	1 (5.2%)	3 (16.7%)	0.26
5 < degree (cases.%)	0 (0%)	0 (0%)	

Values are expressed as mean ± standard deviation except the incidence of knee extension deficiency. *ACL* anterior cruciate ligament

Mann-Whitney U-test

2 X 2 Chi squared test

### Radiological results

Concerning the femoral tunnels in Group A, the incidences of AM and PL tunnel enlargement were 47.4 and 52.6% in the A-P and lateral views, respectively. In Group B, the incidence of AM tunnel enlargement was 33.3% in the A-P view and 50.0% in the lateral view, while the incidence of PL tunnel enlargement was 38.9% in the A-P view and 44.4% in the lateral view (Table 3). No significant differences were observed.

**Table 3:** The incidence of femoral bone tunnel enlargement at 12 monthes postoperatively

	Group A	Group B	*P* value
Anteromedial bundle			
Anteroposterior view (cases. %)	9/19 (47.4%)	6/18 (33.3%)	0.38
Lateral view (cases. %)	10/19 (52.6%)	9/18 (50.0%)	0.87
Posterolateral bundle			
Anteroposterior view (cases.%)	9/19 (47.4%)	7/18 (38.9%)	0.6
Lateral view (cases.%)	10/19 (52.6%)	8/18 (44.4%)	0.62

2 X 2 Chi squared test

The magnitude of percent femoral bone tunnel enlargement of AMB, there was no significant difference between the both groups in the A-P and lateral views. On the other hand, group A showed significantly greater PLB tunnel enlargement than group B (A-P view *p* = 0.01, lateral view *p* = 0.03, respectively) (Table 4).

**Table 4:** Magnitude of bone tunnel enlargement at 12 months after surgery compared to original size

	Group A	Group B	*P* value
Anteromedial bundle			
Anteroposterior view (mean, %)	113 ± 12.1	110 ± 18.9	0.42
Lateral view (mean, %)	121 ± 19.8	115 ± 17.3	0.47
Posterolateral bundle			
Anteroposterior view (mean, %)	126 ± 18.2	113 ± 14.1	0.01*
Lateral view (mean, %)	131 ± 16.2	121 ± 10.1	0.03*

Values are expressed as mean ± standard deviation

Mann-Whitney U-test

*Significant difference between Group A and Group B (*P* < 0.05)

## Discussion

One of the most important findings of the present study was that a high incidence of postoperative femoral tunnel enlargement occurred not only in the 1-week non-weight-bearing group, but also in the 2-week non-weight-bearing group without significant differences. Another important result of the present study was the significant differences in the magnitude of bone tunnel enlargement; the 1-week non-weight-bearing group showed a significantly greater value compared with the 2-week non-weight-bearing group in the PLB both in the A-P and lateral views 12 months after surgery. The information from the present study is of clinical value to identify the early weight-bearing protocol after double-bundle ACL reconstruction with hamstring grafts may induce postoperative femoral bone tunnel enlargement.

Despite of single- or double-bundle, several previous studies have been reported the incidence of femoral bone tunnel enlargement following ACL reconstruction with hamstring grafts [14–30]. A longer postoperative immobilization period did not reduce the incidence of femoral bone tunnel enlargement, but it induced the harmful effect such as the significant loss of postoperative muscle strength [27]. There have been many studies of the relationship between the postoperative immobilization period and bone tunnel enlargement, but few studies evaluated the impact of the postoperative non-weight-bearing period on bone tunnel enlargement. Moreover, the previous reports may have enrolled to the study which cases of ACL reconstruction with meniscal repair or recurvatum. These cases may explain the difference in the outcome because of providing another protocol for immobilization and non-weight-bearing period. Therefore, ACL reconstruction with meniscal repair or recurvatum cases were excluded from the present study, completely.

The pathomechanism of tunnel enlargement is multifactorial and, therefore, not yet fully clarified. Several possible factors associated with bone tunnel enlargement and ACL reconstruction with soft tissue grafts were suggested, previously. Most factors were categorized for two broad pillars, such as biomechanical and biological factors. Among the biomechanical factors related to the micro-motion of the graft within the bone tunnel wall are the bungee cord effect associated with longitudinal graft motion, and the windshield wiper motion effect associated with transverse graft motion, both of which are well known. On the other hand, several biological factors related to bone resorption have been suggested: synovial fluid infiltration to the bony tunnels; foreign body immune response; a nonspecific inflammatory response caused by localized bone necrosis due to thermogenic effects with the drilling process; and biochemical mediators [19–21, 34, 35].

The ACL is the important factor for controlling the tibial motion produced by an applied axial tibial force. While this seems like common sense, it is difficult to prove this phenomenon in the in vivo situation including weight-bearing. Previously, Meyer and Haut demonstrated the ability of joint compression forces to produce ACL ruptures [36, 37]. They reported that application of a high compressive force to an intact knee caused the tibia to displace anteriorly and rotate internally. Markolf et al. reported that they measured tibial motions and ACL forces generated by an axial tibial force application to the knee joint [38]. They concluded that an axial tibial force application to the knee joint induced anterior tibial displacement due to the produced torque with internal and valgus rotations of the tibia. These tibial motions were restrained by ACL function. The following would be a possible scenario: weight-bearing after surgery contributes to the application of an axial tibial force to the knee; the ACL graft affect high pressure to the bone tunnel wall to prevent or restrain the displacement, and, finally, bone tunnel enlargement occurs.

Previously, arthroscopic and clinical findings following ACL reconstruction were showed satisfactory outcome both in accelerated and less-aggressive rehabilitation protocols [39–41]. The present study suggested that a shorter non-weight-bearing period, such as 1 week, after double-bundle ACL reconstruction with hamstring grafts is associated with the potential risk of postoperative femoral bone tunnel enlargement in the PLB. Others, there were no significant differences clinically; general clinical outcome scores, muscle strength, and side-to-side difference between the two different non-weight-bearing periods. Thus, based on these results, a 2-week non-weight-bearing period after surgery might have no significant harmful effect. However, there were also a few cases of slight extension deficiency in both groups without significance. It was unclear that the cause of extension deficiency was due to non-weight-bearing period or effect of knee bracing.

Previously, bone tunnel enlargement was already shown in the first 6 weeks [42], first 3 months [43], and first 6 months [44]. Moreover, this enlargement phenomenon was also reported to progress until 6 months postoperatively [45], and then bone tunnel enlargement stopped progressing. Recently, Shimizu et al. reported that bone tunnel enlargement already started in the first 2 weeks after surgery [46]. These findings indicated that the most important factor is how to control the first 2 weeks of the rehabilitation protocol after surgery including the weight-bearing protocol.

### Limitations

Several limitations must be considered with respect to the present study. First, the small sample size in each group. Second, the present study was conducted without randomization. Third, for bone tunnel size measurement, computed tomography (CT) was not employed. Recently, it has been reported that CT scans may be the most suitable tool to evaluate bone tunnel size [47]. CT scans could be more sensitive for detecting the early bone tunnel findings. However, digital radiography was shown to provide the satisfactorily ability to clarify the signs of bone tunnel enlargement as CT were also reported [17, 48]. On the other hand, CT scans required a high cost, and radiation exposure is an issue for patients. Therefore, computed digital radiography may provide a time- and cost-effective values and decreased radiation exposure to evaluate bone tunnel enlargement than CT scan. Fourth, tibial bone tunnel measurement was not performed in the present study. Postoperatively, it was difficult to determine the each tibial tunnel diameter due to the overlapped two tibial intra-articular outlets in the images. [21]. Fifth, the authors did not determine the inter- and intra-observer variation for measuring the radiographic values. Sixth, the details of the pathomechanisms are still unclear. Seventh, the relation of non-weight-bearing and loss of bone mineral density was not evaluated. Loss of bone mineral density may be one of the big issues of bone tunnel enlargement. However, in spite of these limitations, the present study may contribute to provide the important information on the postoperative non-weight-bearing period.

## Conclusions

The present prospective, clinical and radiographical investigation provided that early weight-bearing protocol after double-bundle ACL reconstruction with hamstring grafts might have the significant potential risk for development of postoperative femoral bone tunnel enlargement of the PLB. There was no significant difference in the clinical outcomes between 1- and 2-week postoperative non-weight-bearing periods. To reduce and prevent the femoral bone tunnel enlargement, the comprehensive management could be considered and required to establish the suitable early stage rehabilitation protocol after surgery.

## Data Availability

The datasets used and/or analyzed during the current study available from the corresponding author on reasonable request.

## Abbreviations

ACL: Anterior cruciate ligament
AMB: Anteromedial bundle
A-P: Anteroposterior
BTB: Bone-patellar tendon-bone
CT: Computed tomography
MRI: Magnetic resonance imaging
PLB: Posterolateral bundle
